# Electrical Breakdown Mechanism of ENB-EPDM Cable Insulation Based on Density Functional Theory

**DOI:** 10.3390/polym15051217

**Published:** 2023-02-28

**Authors:** Zhiyi Pang, Yi Li, Yiyi Zhang

**Affiliations:** 1 Faculty of Intelligent Manufacturing, Nanning University, Nanning 530200, China; 2 School of Mechanical and Electrical Engineering, Liuzhou Vocational & Technical College, Liuzhou 545000, China; 3 School of Electrical Engineering, Guangxi University, Nanning 530004, China

**Keywords:** electrical insulation, external electric field, density functional theory, molecular simulation

## Abstract

The ethylene propylene diene monomer (EPDM) is utilized in high voltage direct current (HVDC) cable accessories due to its exceptional insulation properties. The microscopic reactions and space charge characteristics of EPDM under electric fields are studied using density functional theory. The results indicate that as the electric field intensity increases, the total energy decreases while the dipole moment and polarizability increase, leading to a decrease in the stability of EPDM. The molecular chain elongates under the stretching effect of the electric field and the stability of the geometric structure decreases, resulting in a decline in its mechanical and electrical properties. With increased electric field intensity, the energy gap of the front orbital decreases, and its conductivity improves. Additionally, the active site of the molecular chain reaction shifts, leading to different degrees of hole trap and electron trap energy level distribution in the area where the front track of the molecular chain is located, making EPDM more susceptible to trapping free electrons or injecting charge. When the electric field intensity reaches 0.0255 a.u., the EPDM molecular structure is destroyed, and its infrared spectrum undergoes significant changes. These findings provide a basis for future modification technology, and theoretical support for high voltage experiments.

## 1. Introduction

A high voltage direct current (HVDC) cable transmission system possesses several advantages, including low cost, reactive power compensation, and compatibility [1], so as to solve the interconnection problem between the new energy power generation system and the alternating current (AC) backbone grid [2]. As a critical component of the smart grid and future global energy interconnection grids, the HVDC cable is deemed to be an indispensable aspect of the direct current (DC) power grid [3]. The utilization of extruded polymer insulated DC cables in new energy grid-connected projects that employ flexible DC transmission technology is driven by the cables’ superior electrical performance, weather resistance, and thermal stability in environments of high humidity, strong electric fields, and temperature fluctuations [4]. Currently, the insulation materials utilized in extruded HVDC cables primarily consist of low density polyethylene (LDPE) [5], high density polyethylene (HDPE) [6], crosslinked polyethylene (XLPE) [7], ethylene propylene rubber (EPR) [8], and ethylene polypropylene (PP) [9].

In the pursuit of advancements in high voltage grade transmission lines, the design and implementation of high voltage cable accessories constitutes a crucial aspect [10]. Studies have indicated that insulation failure of cable accessories is a leading cause of frequent faults in DC cable lines [11]. HVDC cables need to withstand a long-term unipolar strong electric field [12], which makes the insulation material age, and undergo partial discharge and even insulation breakdown [13]. Currently, EPDM is widely utilized as the main insulating material for HVDC cable attachments due to its low dielectric loss, resistance to partial discharge, and resistance to inter-molecular ionization [14]. EPDM cables have been applied in the insulation and shielding of mining, nuclear power, and marine cables, and they have demonstrated a service life that is 10 times longer than other rubbers [15]; meanwhile, in terms of ultra-high voltage leads and wiring, EPDM insulated cables cannot be replaced by other rubbers [16]. With advancements in rubber processing technology, the reliability of EPDM in HVDC cable accessories continues to improve. A study conducted by DU investigated the growth characteristics of EPDM electrical branches under different tensile and compression rates [17], finding that the length and cumulative damage failure area increased with an increase in tensile rate, and decreased with an increase in compression rate [18]. DU find that the addition of polyhedral oligomeric silsesquioxane (POSS) to EPDM could inhibit electrical branches well [19]. Furthermore, SU’s study demonstrated that the synergistic effects caused by EPDM electrical branches can be inhibited through the rational combination of main and auxiliary antioxidants [20]. The internal insulation of EPDM materials is vulnerable to breaking under the prolonged effect of electric fields, leading to the accelerated aging of the insulation [21,22]. Hence, investigating the structure and properties of EPDM under external electric fields is of immense significance.

Molecular simulation technology, as an emerging research tool, offers a unique capability to simulate and to calculate experiments that are impossible to achieve under real experimental conditions [23]. Furthermore, it provides insights into the underlying mechanisms of physical and chemical microscopic processes, and in some instances, it may even result in more accurate calculations compared to experimental measurements [24]. Now, the molecular simulation method has been widely used in the study of insulating oil [25] and insulating paper aging [26]. Density functional theory, a quantum mechanical-based electronic structure theory [27], is commonly used to study multi-electron systems by utilizing electron density as the basic parameter, rather than wave function. This method finds widespread applications in the fields of physics, chemistry, and materials science, particularly in the area of molecular and condensed matter research. The molecular simulation method presents promising prospects for a wide range of applications, and provides a new avenue for investigating the thermal aging and electrical breakdown mechanisms of insulation under electric fields.

In order to obtain the weak sites inside the material, and to provide theoretical support for high-voltage experimental evidence, and avoid blindly adding electric fields in high-voltage experiments, this study uses quantum chemistry methods for theoretical research. The method studied in this paper is based on density functional theory. The process begins with the establishment of an EPDM molecular model and its geometric optimization, which is carried out using the Gaussian 09W software. Subsequently, the electrostatic potential surface of the EPDM molecule is analyzed through the use of Multiwfn [28] software, in order to identify the internal active reaction sites. The microstructure, total energy, dipole moment, and polarizability of the EPDM molecules are calculated under various external electric fields. Finally, the electric breakdown mechanism of EPDM when subjected to electric fields is thoroughly analyzed by examining the infrared spectrum and space charge characteristics of the EPDM molecules.

## 2. Methods

### 2.1. Model Construction

**Figure 1:**
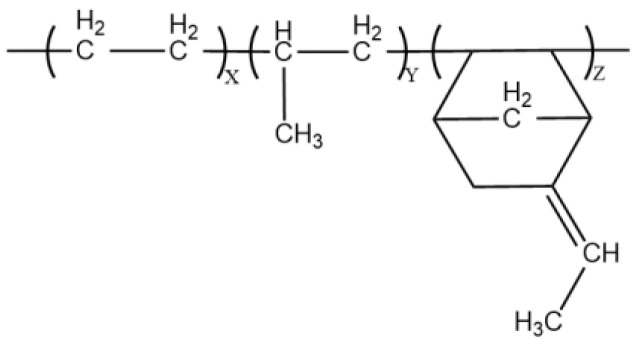
The molecular structure of ENB-EPDM (Ethylene-propene-ethylene-norbornene).

In this experiment, the ethylene propylene diene monomer (EPDM) material utilized is composed of ethylene, propylene, and a third monomer, namely ethylene-norbornene (ENB), with an unsaturated olefin bond on its side chain that accelerates the vulcanization rate. The vulcanized EPDM boasts high strength, filling, and extrusion performance [29], and is commonly utilized in the production of molded products such as sealing strips, rubber tubes, and electrical wires and cables. Its monomer molecular structure is shown in Figure 1. In this study, a semi-empirical method is used to solve the simplified Schrodinger equation, which can describe the electron distribution, molecular structure, and properties. Its electrical and mechanical properties make it an ideal insulating material for high-voltage DC cable accessories. Semi-empirical methods play an important role in analyzing molecular structures, and they are suitable for molecular structures such as ENB-EPDM in this study. Models with different DPs were selected for pre-simulation as shown in Table 1, and the results showed that the degree of polymerization had little effect on the results. Thus, only a single ENB-EPDM molecule was selected for in-depth computational analysis while still meeting the accuracy requirements. This paper aims to construct the model of the ENB-EPDM molecule and simulate its microscopic mechanism under the influence of electric fields.

### 2.2. Theoretical Calculations and Methods

The Hamiltonian *H* of ENB-EPDM molecules under an external electric field can be expressed as Equation (1). *H*_0_ is the Hamiltonian of the molecule without an electric field; *H*_*int*_ is the Hamiltonian when the external electric field interacts with the molecular system. In the case of dipole approximation, the interaction energy between the molecular system and the external electric field can be expressed as Equation (2). *μ* is the electric dipole moment and *F* is the external electric field force.

(1) $$H=H_{0}+H_{int}$$

(2) $$H_{int}=-\mu \times F$$

**Figure 2:**
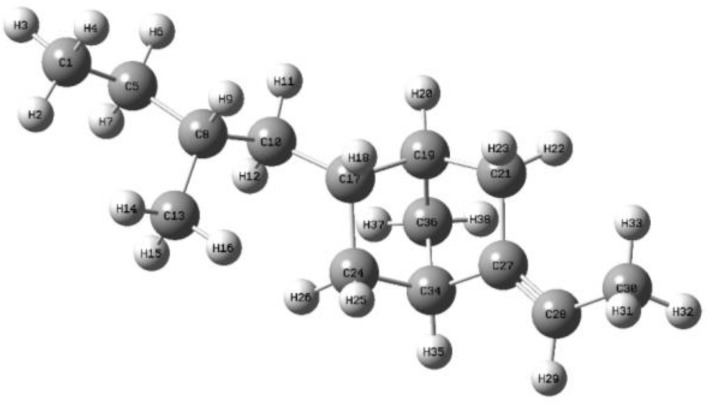
Monomeric molecular model of EPDM.

The molecular dynamics method is used to simulate the evolution of an electronic structure, and the molecular core skeleton of ENB-EPDM. Specific steps: The B3LYP functional [30] and 6-31G* basis sets [31] based on density functional theory [32] are used to optimize the geometric configuration of the ENB-EPDM molecular model to obtain the stable conformation of a molecule with the lowest energy [33]. Neutral and singlet charges and spin states are assumed for the compound studied, and the unrestricted Kohn-Sham formalism is used in the calculation. The optimized molecular model is shown in Figure 2, where the gray represents a carbon atom C, and the white represents a hydrogen atom H. Its total energy is at its lowest, there is no imaginary frequency, and the results of Maximum Force, RMS Force, Maximum Displacement, and RMS Displacement in the calculation result all converge. At present, the voltage level of the transmission grid is becoming higher, and the requirements of the insulation materials are gradually increasing. Therefore, when extreme power failure occurs, the electric field generated may exceed the air breakdown electric field. The same method and base group are used to apply an electric dipole field in the X direction from 0 to 0.0255 a.u. (1 a.u. = 5.142 × 10^11^ V/m) of the molecular chain, respectively, and the optimization and single point energy calculation are conducted to study the microscopic mechanism of the electrical aging of the ENB-EPDM materials. All simulation calculations are carried out in the Gaussian 09W software package. Multiwfn 3.7 software [34] and VMD 1.9.3 [35] software are used for further analysis.

## 3. Simulation Results and Discussion

### 3.1. Effect of an External Electric Field on Molecular Dipole Moment and Energy

**Figure 3:**
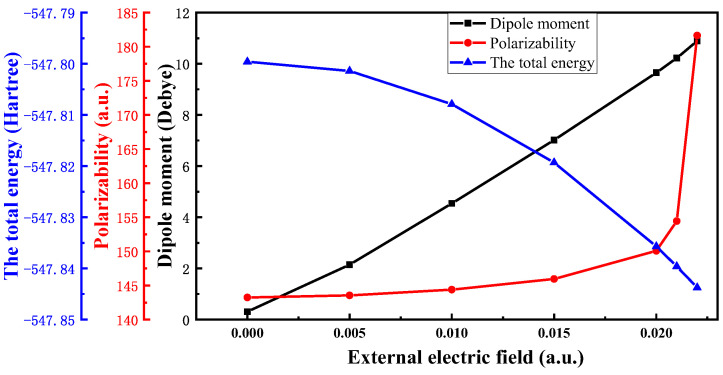
The variation of the total energy, dipole moment, and polarizability.

Figure 3 shows that the total energy of a molecular system gradually decreases with the increase in the electric field intensity, and the decrease is more and more large, because the electron transfer occurs along the electric field direction, making the charge on each atom in the electric field direction larger, and then increasing the electric dipole moment *μ* of the ENB-EPDM molecule. Equation (2) shows that with the increase in the electric field intensity, the absolute value of electric field and molecular interaction energy *H*_*int*_ increases, and its interaction energy is negative, resulting in a decrease in the total energy *H* of the molecular system. Figure 3 shows the change of the dipole moment of the ENB-EPDM molecule under an external electric field. The dipole moment increases with the increase in the electric field intensity, which indicates that with an increase in the X-axis forward electric field intensity, the molecular polarity becomes larger. The molecular polarizability of ENB-EPDM varies with the electric field intensity, as shown in Figure 3. The degree of polarizability reflects the degree of difficulty for the dielectric to polarize under the action of an electric field. A high polarizability denotes that the dielectric polarization occurs easily, on the contrary, and a small polarizability denotes that it is difficult for the dielectric to polarize. The corresponding macroscopic phenomenon is that the polarization strength of the cable material increases. An ultrahigh electric field intensity leads the dielectric polarization seriously. When the applied electric field reaches a certain value, the bound charge in the dielectric material of the cable will break out of the molecular range and become free charge, thus making the dielectric material lose insulation performance.

### 3.2. Effect of the External Electric Field on the Geometry of the Molecule

**Figure 4:**
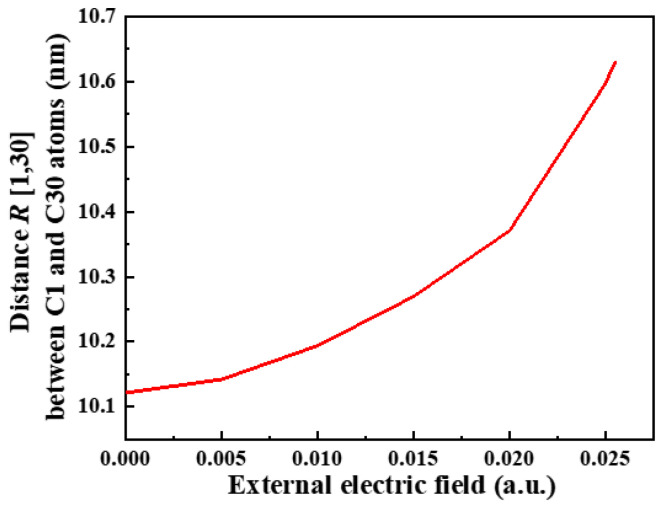
The trend of distance *R* [1, 30] between the C1 and C30 atoms.

**Figure 5:**
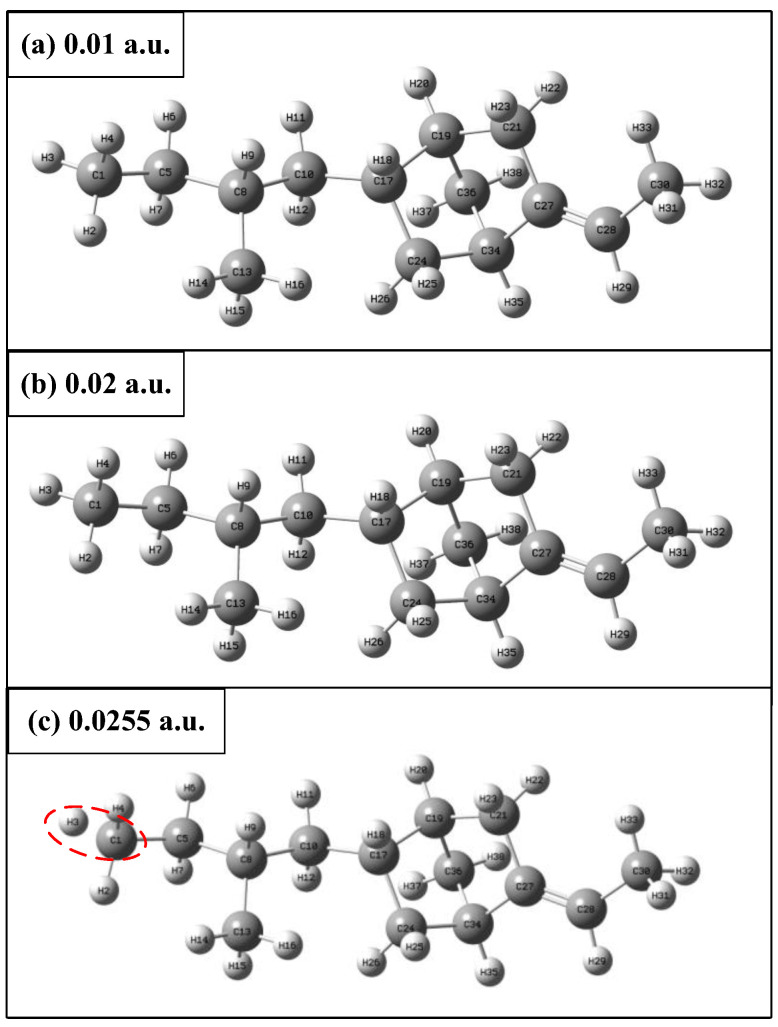
ENB-EPDM molecule at different external electric fields.

After optimizing the ENB-EPDM molecule to obtain a stable spatial structure, the variation trend of distance *R* [1, 30] between the leftmost C1 atom and the rightmost C30 atom of the molecular chain under different external electric fields (0~0.0255 a.u.) is shown in Figure 4, which gradually becomes larger with the increase in the electric field intensity. Due to the effect of an electric field, the positive and negative charges transfer in the molecular system, which produces a certain stretch effect on the molecular chain and reduces the stability of the molecular geometric structure. The dynamic changes of the ENB-EPDM molecular chain under different electric field intensities are shown in Figure 5, where the direction of the applied electric field is the trend of the molecular chain extension. When the electric field intensity reaches 0.0255 a.u., the C-H bond of the ENB-EPDM molecule is broken, as shown in Figure 5c.

### 3.3. Effect of an Electric Field on the Space Charge Properties of ENB-EPDM

Under the long-term polarization of electric field, the geometry of the ENB-EPDM molecular chain will be gradually stretched. When the internal field strength reaches a certain value, the molecular chemical bond will break, resulting in the breakdown of insulation. The space charge distortion in the molecular chain under the continuous action of the electric field is the main reason for the increase in the internal field intensity in the insulating material.

**Figure 6:**
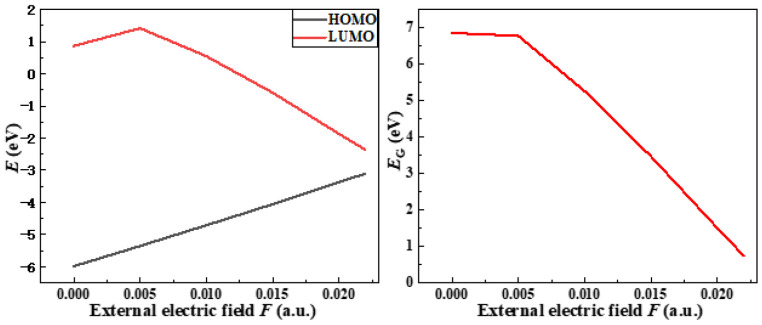
The variations of molecular frontier orbital energy level and energy gap with electric field intensity.

According to the frontier orbital theory, the energy of the highest electron occupied orbital (HOMO) in a molecule is the highest, the electron is the least bound, and the transition is the most likely to occur. The lowest unoccupied orbital (LUMO) has the lowest energy of all unoccupied orbitals and accepts electrons the most easily. The energy gap (Eg) between the HOMO and the LUMO of the front orbital is a measure of the electron’s ability to move from an occupied orbital to an empty one, and it is often used to measure the molecule conductivity. The smaller Eg is, the better the conductivity of the molecule is, and the easier it is for electrons to transition from the HOMO to LUMO orbitals. As shown in Figure 6, the HOMO orbital energy level is −5.99 eV and the LUMO orbital energy level is 0.87 eV without an external electric field. When the electric field intensity reaches 0.022 a.u., the HOMO orbital energy level becomes −3.11 eV, and the LUMO orbital energy level becomes −2.37 eV. At the same time, Eg between the HOMO and LUMO orbitals is constantly decreasing, so that the electrons bound in the molecular orbital are easier to transition from the HOMO orbital to the LUMO orbital, and the conductivity of the molecule is becoming better and better. When Eg is reduced to a certain value, the electrons can move freely inside the material to form a current. Atoms or groups at the locations of HOMO and LUMO orbitals have the highest reaction activities, and are more likely to react with other molecules or free radicals, resulting in the breakage of molecular chemical bonds and the disintegration of molecular chains, resulting in the decline of insulating properties of insulating materials.

**Figure 7:**
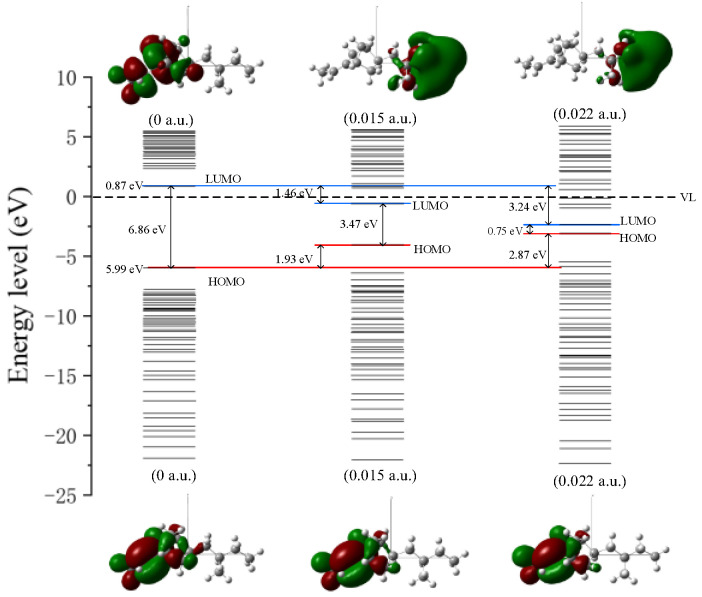
The energy level distribution and the frontier orbit of ENB-EPDM.

In order to analyze the microscopic variation characteristics of the space charge inside the ENB-EPDM under the continuous electric field, the changes of trap energy level and the orbital cloud map can be used to discuss. It can be seen from the distribution of the molecular orbits (MOs) map that before the electric field is applied, the HOMO orbital is mainly concentrated to the left end of the molecular chain, which is easily attacked by electrophile reagents and electrophilic reactions that occur. Additionally, the LUMO orbital is mainly concentrated to the left end of the molecular chain, which is vulnerable to nucleophile attack and nucleophilic reaction. However, the effect of the external electric field makes the distribution of the molecular front track change obviously, and the reaction active site of molecule also changes accordingly, as shown in Figure 7. At the electric field strength of 0.015 a.u., LUMO orbital shifts in the opposite direction. When the electric field reaches 0.022 a.u., the LUMO orbital moves towards the end of the molecular chain. Thus, electrophilic and nucleophilic activities are shown on the left and right sides of the molecular chain, respectively. This phenomenon indicates that the applied electric field leads to the continuous decrease in the LUMO orbital energy level, and the continuous increase in the HOMO orbital energy level, and it also changes the active site of the molecular chain reaction.

As can be seen from the energy level distribution diagram in Figure 7, the molecular orbital energy gap can reach 6.86 eV before the electric field is applied, while the energy gap width of the insulator is generally larger than 4.5 eV. When the continuous action of the electric field reaches 0.022 a.u., the change of energy level distribution is more obvious, and the HOMO and LUMO orbital energy levels are very close; Eg between the highest electron occupied orbital and the lowest empty orbital is only 0.75 eV. At this time, the insulating paper will be in the semiconductor state. It indicates that with the continuous action of electric field, the electron trap energy level will become larger and larger, and the ability of molecular orbitals to capture free electrons will become stronger. At the same time, the energy level of the hole trap will become larger and larger in the position of the HOMO orbital where the electrons are more active. The HOMO orbital is related to the top position of the valence band, and the LUMO orbital is related to the bottom position of the conduction band. Under the long-term action of the electric field, the HOMO orbital energy level gradually increases and the LUMO orbital energy level gradually decreases, which makes the frontier orbital energy gap gradually decrease, and the molecular conductivity goes up. When the energy gap is reduced to a certain value, the molecular chemical bond will break. As a result, the insulating properties of the material decrease.

**Figure 8:**
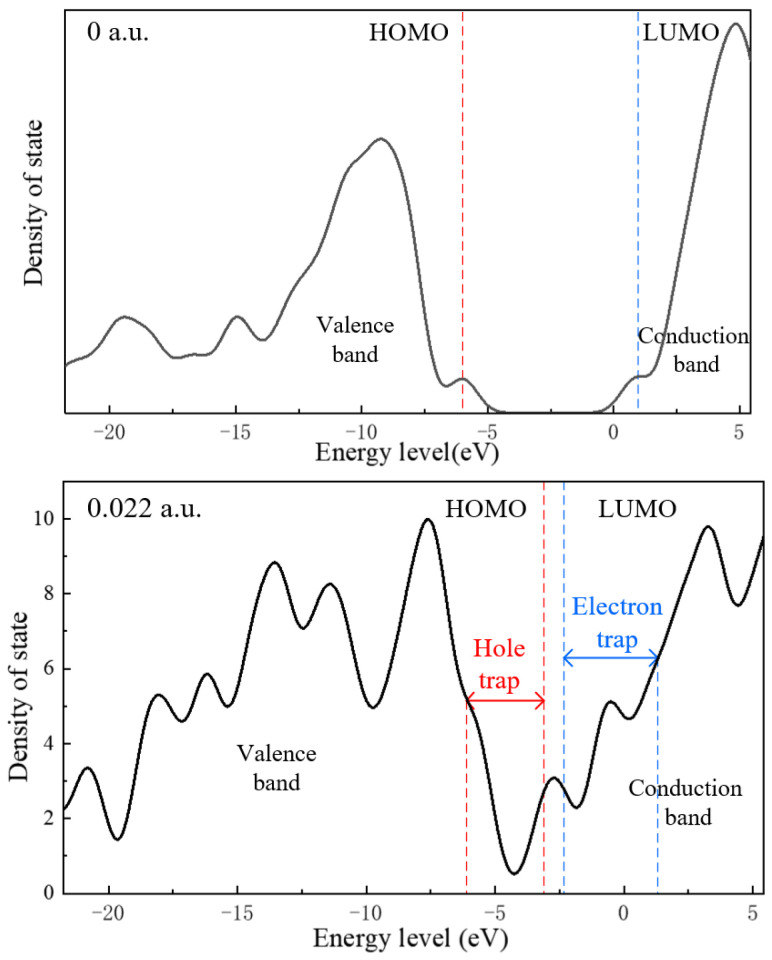
The density of states at 0 a.u. and 0.022 a.u.

The results calculated using the Gaussian software are then processed using Multiwfn software using the default parameters, with each orbital having a strength value of 1, and the degeneracy using the default 0.005 eV threshold. Figure 8 shows the molecular chain of ENB-EPDM before the electric field is applied, and the electric field strength is 0.022 a.u. From the density of states (DOS) distribution, it can be seen that under the action of an electric field, a certain amount of deep trap and shallow trap energy level distribution appears near the valence band and the conduction band of the ENB-EPDM molecular chain. With the continuous action of an electric field, the density of trap energy levels formed by molecular orbitals will further increase. The HOMO orbital energy level shifts to a higher energy level, which introduces more hole traps near the valence band. The LUMO orbital energy level moves to the lower energy level direction, more electron traps are introduced near the conduction band, and the number of electron traps is more than the number of hole traps. The difference between the number of holes and electron traps indicates that the effect of the electric field will make the molecular chain of the ENB-EPDM more likely to capture free electrons or injected charges, resulting in a distortion of local field strength inside the material.

**Figure 9:**
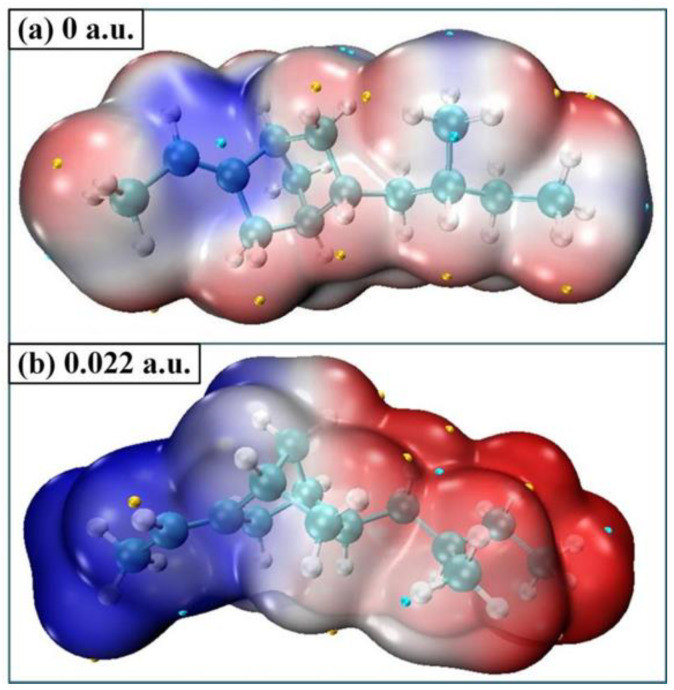
Molecular electrostatic potential surface at 0 a.u. and 0.022 a.u.

Figure 9 shows the surface electrostatic potential distribution of ENB-EPDM molecules, which can be used to predict the reaction active site. The atoms corresponding to the positions with more negative electrostatic potential on the molecular surface are more likely to have electrophilic reactions, and the atoms corresponding to more positive positions are more likely to have nucleophilic reactions. The depth of color on the electrostatic potential map of the molecular surface reflects the strength of the electrostatic potential. The positive electrostatic potential region is represented by red, and the negative electrostatic potential region is represented by blue. The yellow sphere corresponds to the point of maximum electrostatic potential, and the green sphere corresponds to the point of minimum electrostatic potential. It can be seen from the molecular electrostatic potential surface that the global minimum value of the electrostatic potential of the molecular surface is near the C=C bond of the carbon ring, showing a relatively strong degree of electronegativity, with more surrounding electrons and a higher density, which has electrophilic activity and is prone to electrophilic reactions. The global maximum value of the electrostatic potential on the molecular surface is near the hydrogen atom on the surface, indicating that this region has a high degree of nucleophilicity and is prone to nucleophilic reactions. When the electric field intensity reaches 0.022 a.u., the electrostatic potential distribution of the molecular surface shows a great difference, and the positive electrostatic potential and the negative electrostatic potential are displayed on the left and right sides of the chain, respectively. The above results can be speculated that when the ENB-EPDM molecules are subjected to an external electric field, the chemical bond is broken at the maximum or minimum electrostatic potential due to its strong reaction activity. When the insulating material is subjected to an extreme field strength, the chemical bond in the weak position inside the material gradually breaks, which causes the change of the dielectric constant of the material, and finally the overall insulation performance of the material decreases, breaking through the insulation level of the electrical material, resulting in electrical breakdown. In electrical engineering, even if the insulation level of the insulating materials decreases at one location, it is easy to cause electrical failure and electrical breakdown without waiting until the overall insulation level of the insulating materials decreases.

### 3.4. Molecular IR Spectra under Different Electric Field Intensities

**Figure 10:**
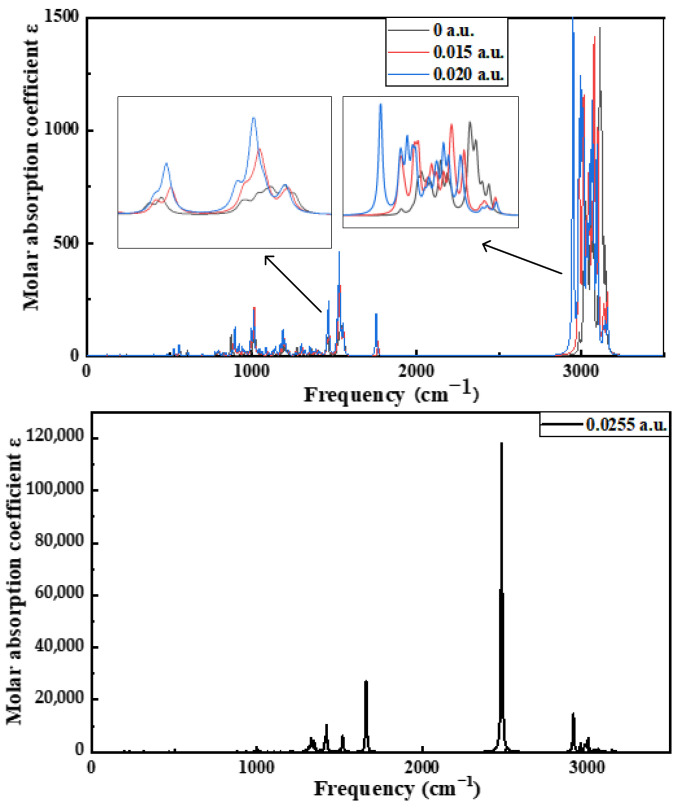
Infrared spectra of ENB-EPDM molecules with different electric field intensities.

Finally, Multiwfn software is used to further process the results calculated using Gaussian software. The default parameter is used, and the frequency correction factor is 0.9614. The infrared spectra obtained via a simulation of the ENB-EPDM molecules at different electric field intensities are shown in Figure 10. Under the electric field of 0~0.02 a.u., the infrared spectrogram does not change greatly, indicating that the low electric field intensity has little influence on the structure of the ENB-EPDM molecules, and that the molecular system still maintains its structural stability. However, under the electric field of 0.0255 a.u., the infrared spectrograms have a large difference, and the main difference is that the peak value of the absorption peak fluctuates greatly, the infrared activity of the vibration mode in more intervals is significantly enhanced, the peak value of the corresponding absorption peak is significantly increased, and the C-H stretch vibration at 2555.15 cm^−1^ is the strongest at the end of the molecular chain. With the increase in the external electric field, the corresponding absorption peak appears to split. This indicates that the nuclear skeletons and electronic structures of the ENB-EPDM molecules have changed greatly under the action of a strong electric field, which leads to the enhancement of the infrared activity of vibration modes in more intervals, and the shift of resonant frequency. When the electric field intensity continues to increase, the virtual frequency appears in the infrared spectrum of the ENB-EPDM molecule, indicating that the molecular structure is not stable. In this unstable state, the structure of the ENB-EPDM molecule is easily destroyed. The inflection point where the internal molecular structure of the ENB-EPDM dielectric changes is the starting point of the electrical branch aging of the insulating materials. The internal molecular structure of ENB-EPDM is destroyed, and a large number of free radicals are formed. Free radicals will attack groups on other chains, resulting in bond breaking and the cracking of more molecular chains. The breakage of the molecular chain must lead to the destruction of the ENB-EPDM material, thus reducing the electrical strength of the cable, and finally the polymer insulation breakdown phenomenon. It is of great significance for studying the mechanism of electrical aging.

## 4. Conclusions

In this study, the molecular model of ENB-EPDM was constructed using a molecular simulation approach based on quantum mechanics to analyze and to calculate the influence of external electric fields on the internal microscopic characteristics of ENB-EPDM molecules. The results reveal changes in the insulation properties of ENB-EPDM materials at the molecular level. The key findings are:

(1) The electrostatic potential surface analysis of ENB-EPDM molecules shows that the C=C bond and the H atoms near the carbon ring have strong reactivity and are prone to reaction, providing evidence for the initial bond breaking of ENB-EPDM under strong electric field conditions, and a theoretical basis for modifying ENB-EPDM insulation materials.

(2) With an increasing external electric field, the dipoles of the ENB-EPDM molecules will rotate in a directional manner, forming equivalent polarized space charges inside the ENB-EPDM molecules. The stronger the electric field, the higher the polarization rate and space charge density, leading to an increase in molecular activity and instability, or even the destruction of the molecular spatial structure. The analysis of the space charge characteristics shows that the energy gap decreases, leading to better molecular conductivity. When the energy gap decreases to a certain level, the chemical bond breaks. The electric field changes the reactive sites of the molecular chain, leading to electrophilic and nucleophilic activities at both ends of the chain, and the uneven distribution of trap energy levels along the front track of the molecular chain, resulting in a distorted local field strength inside the molecule.

(3) The inflection point in the molecular structural characteristics is the starting point of electric branch formation. When the electric field intensity reaches 0.0255 a.u., ENB-EPDM molecules undergo electrical breakdown, with the hydrogen atom at the end of the molecular chain breaking first and generating free radicals, which in turn affects the internal microscopic characteristics of ENB-EPDM. This results in changes in the corresponding infrared spectrogram, providing a theoretical basis for evaluating the state of insulation. This is due to the increasing voltage level of the transmission network, the high requirements for insulation materials, and the breakdown of the ultra-high voltage of power failures. Based on the above situation, molecular simulation can provide theoretical support for the subsequent engineering practical tests of this project. It is targeted and avoids blindly carrying out experiments. Through the theoretical research of molecular simulation, it will be able to save the considerable costs of a strong electric field. It can also provide support for our subsequent material modification technology.

## Figures and Tables

**Table 1:** Breakdown of molecules with different DPs under electric field (EF).

EF (a.u.)	Monomer	Dimer	C1	Monomer	Dimer
0	No	No	0.006	No	No
0.001	No	No	0.007	No	No
0.002	No	No	0.008	No	No
0.003	No	No	0.009	No	No
0.004	No	No	0.010	No	No
0.005	No	No	0.011	No	No

## Data Availability

The data that support the findings of this study are available from the cor-responding author upon reasonable request.
